# High Bed Occupancy Rates in Internal Medicine Departments Are Associated with Lower Hand Hygiene Compliance

**DOI:** 10.3390/medicina62010137

**Published:** 2026-01-09

**Authors:** Adi Saad, Oryan Henig, Ruth Sasportas, Gil Fire, Tomer Ziv-Baran

**Affiliations:** 1 Department of Epidemiology and Preventive Medicine, School of Public Health, Gray Faculty of Medical & Health Science, Tel Aviv University, Tel Aviv 69978, Israel; 2 Infection Prevention and Control, Tel Aviv Sourasky Medical Center, Tel Aviv 64239, Israel; 3 Hospital Management, Tel Aviv Sourasky Medical Center, Tel Aviv 64239, Israel; 4 Gray Faculty of Medical & Health Science, Faculty of Management, Tel Aviv University, Tel Aviv 69978, Israel

**Keywords:** hand hygiene, bed occupancy, workload, internal medicine departments, quality index

## Abstract

*Background and Objectives:* The growing number of patients seeking medical care in the internal medicine departments over the past decades has been accompanied by an increase in the bed occupancy rate. This is associated with a heavier work burden among the professional staff members, which may lead to a lower quality of care. Therefore, this study aimed to evaluate the association between the bed occupancy rate and staff compliance with hand hygiene regulations. *Materials and Methods:* This ecological study included 9 internal medicine departments (~300 beds) in a single medical center between 01/2017 and 12/2019. Routine hand hygiene performance was evaluated randomly, and the association between the bed occupancy rate and the staff’s compliance with the hospital regulations was studied. Univariate and multivariable analyses were performed by the generalized estimating equation model. *Results:* The study included 12,736 episodes that warranted hand hygiene practices (“opportunities”). The overall hand hygiene performance rate was 78.3% (physicians 76.2%, nurses 80.7%, and healthcare assistants 76.9%). There was an approximately 2% decline in staff compliance for each 10% increase in bed occupancy rate (adjusted IRR 0.98, 95%CI 0.97–0.99, *p* < 0.001). Stratification by staff members showed a significant decline in routine hand hygiene practices among physicians (adjusted IRR 0.97, 95%CI 0.95–0.99, *p* < 0.001) and healthcare assistants (adjusted IRR 0.97, 95%CI 0.96–0.99, *p* < 0.001) but not among nurses (adjusted IRR 0.99, 95%CI 0.98–1.01, *p* = 0.392). *Conclusions:* An increase in bed occupancy rate is associated with a decrease in the hospital staff’s compliance with hand hygiene and therefore may lead to a lower quality of care.

## 1. Introduction

Internal medicine departments are pivotal to the care of patients with acute or deteriorating chronic conditions [1]. Longer life expectancies have led to a notable rise in the number of patients with complex medical needs seeking care within these departments [1,2,3]. However, this surge in patient complexity and volume has not been accompanied by either a proportional increase in available hospital beds or in professional personnel. As a result, the workload of the medical and nursing staff within these departments has increased [2,4,5,6].

Stang et al.’s systematic review on workload measures associated with the quality of care in emergency departments (ED) showed that the bed occupancy rate is one of the top 3 measures associated with the quality of care [7]. The quality of care is assessed through indicators designed to enhance medical service quality for patients [8]. One key indicator used in hospitals is healthcare staff’s adherence to hand hygiene protocols. Several reports have demonstrated that hand hygiene compliance is important for the reduction in transmission of pathogenic microorganisms [9,10,11]. In Luangasanatip et al.’s meta-analysis, all of the included studies reported that improvements in hand hygiene were associated with reductions in hospital-acquired infections and resistance rates [12].

“Bed occupancy” is an administrative measure of workload. It is easy to estimate, and the data are readily available to hospital management. We hypothesized that hospital bed occupancy rates, as an indirect measure of workload, are related to the level of staff compliance with the hand hygiene regulations for controlling the spread of nosocomial infections. Previous studies on such associations had included only limited numbers of episodes that warranted proper hand hygiene practices (“opportunities”), mostly ranging from 800 to 2000 [13,14,15,16,17]. Additionally, those studies included departments other than internal medicine, such as ED, intensive care units, and surgical departments, which harbor innumerable confounders in the attempt to establish straightforward cause-and-effect relationships. Therefore, this study aimed to investigate, for what we believe to be the first time, the association between bed occupancy rates and the staff’s compliance with hand hygiene solely among comparable internal medicine departments in a large municipal medical center.

## 2. Materials and Methods

### 2.1. Study Design

This ecological study was conducted at the Tel Aviv Sourasky Medical Center, a 1500-bed tertiary care institution located in central Israel. The medical center serves a population of one million people and includes 4 hospitals: general, children’s, maternity and women’s, and rehabilitation. The general hospital had 9 internal medicine departments with a total average of 300 beds during the study period. All hand hygiene observations that were performed between January 2017 and December 2019 in all internal medicine departments were included. The findings of the study were based upon data collected according to appropriate queries from the hospital database and from data derived from a routine hand hygiene performance survey that evaluates the compliance of physicians, nurses, and healthcare assistants throughout the medical center [18]. Healthcare assistants were defined as personnel assisting with patient bathing, feeding, and cleaning of the immediate patient environment and shared equipment. All staff members received hand hygiene training through a standardized e-learning module in accordance with Ministry of Health guidelines. In addition, healthcare assistants received in-person instruction, and nurses underwent further training sessions emphasizing hand hygiene practices within the clinical procedures they performed. This retrospective and anonymized study was approved by the local Institutional Review Board (IRB, 0235-21-TLV). Given the ecological nature of the study, only aggregate data were collected. Since no individual data were collected, informed consent was not required.

### 2.2. Study Variables

The hospital bed occupancy rates were measured every day at midnight, following standard national practice. While minor variations occur throughout the day due to patient admissions and discharges, midnight occupancy is generally considered a reliable measure of overall daily hospital workload. Bed occupancy was calculated as the ratio between the actual number of hospitalized patients and the number of beds in each department. The rate of the staff’s compliance with hand hygiene practices was measured based on the observations of infection control nurses and other observers who were trained by the National Infection Control Unit of the Ministry of Health [18]. Each observation session lasts approximately 20 min. The frequency of observations is determined by the hospital’s risk assessment process and ranges from 20 to 50 observations per month across hospital departments. Observations are conducted overtly; therefore, the likelihood of a Hawthorne effect may increase as staff become familiar with the different observers, making it difficult to mitigate this effect. Compliance was evaluated according to the “Five Moments” model, which explains how the medical and nursing staffs should implement proper hand hygiene at the model’s 5 time points: Moment 1—Before touching a patient; Moment 2—Before performing a clean/aseptic procedure; Moment 3—After body fluid exposure risk; Moment 4—After touching a patient; Moment 5—After touching patient surroundings [9].

The compliance rate was calculated as the ratio of the number of performances divided by the total number of opportunities. Age, sex, comorbidity index, functional index, and invasive ventilation data of patients who were hospitalized at the time of the routine hand hygiene performance survey were collected from the patient electronic medical record system. The Charlson Comorbidity Index (CCI) and the Norton scale were used to evaluate the severity of patient comorbidity and functionality, respectively. CCI is an index that estimates the risk of death from comorbid disease. The index gives a weighted score, which predicts mortality within 1 year of hospital admission in patients without trauma. The elaborated score ranges from 0 to 24, where a higher CCI indicates a higher risk of death [19,20]. CCI was demonstrated in a meta-analysis and in other studies from various medical disciplines as being associated with longer length of hospital stay, increased likelihood of in-hospital death, and higher hospital costs [21,22,23,24]. The Norton scale is an index that assesses the risk of pressure ulcer formation and takes into account the criteria of physical condition, mental condition, activity level, mobility level, and control of the sphincters. The score ranges from 20 (minimal risk) to 5 (maximum risk) [25]. The Norton scale has been associated with poor patient outcomes, including mortality, prolongation of hospitalization, and an increase in the incidence of complications during hospitalization [26,27]. The mean age, proportion of females, mean CCI, mean Norton score, and proportion of ventilated patients were calculated. The day of the week was considered a weekday or weekend.

### 2.3. Statistical Analysis

A total of 194 observations were needed in order to identify a weak correlation (r = 0.2) between the proportion of hand hygiene performance and hospital bed occupancy, with a significance level of 5% and a power of 80%. The distribution of the continuous variables was evaluated by a histogram and a Q-Q plot. Variables were described as mean and standard deviation if they were normally distributed, as median and interquartile range if they were non-normally distributed, or as mean and range. Friedman test was used to compare the number of opportunities in each observation between staff categories. Univariate and multivariable negative binomial regressions were used in order to estimate the association between bed occupancy rate to staff compliance. The generalized estimating equations model was applied to analyze the relationship between occupancy rate and compliance while controlling for the repeated measurements within the departments. The natural logarithm (ln) of the number of opportunities was considered the denominator and used as an offset in the model. All statistical tests were 2-sided, and statistical significance was defined as *p* < 0.05. The data were collected in Microsoft Excel and analyzed using SPSS software (IBM SPSS Statistics for Windows, ver. 28, IBM Corp., Armonk, NY, USA, 2021).

## 3. Results

The study spanned 1043 days, during which hand hygiene observations were conducted by infection control nurses and other trained observers. In total, 12,736 hand hygiene opportunities were tallied during the study period. The number of opportunities documented in each observation was significantly different between subgroups (*p* < 0.01), with nursing staff having the highest number (median 5, IQR 3–9), followed by physicians (median 3, IQR 1–5) and healthcare assistants (median 2, IQR 0–3).

The average occupancy rate of beds was 99.0% (SD 14.2), ranging from 55.8% to 135.5%, with a median of 100% (IQR 88.2–110.8%). The male-to-female ratio was approximately 1:1, and the mean age of the patients was 71.6 years. The study included 15 (1.4%) weekends and 1028 (98.6%) weekdays. The departments’ characteristics on the day of observation are described in Table 1.

**Figure 1:**
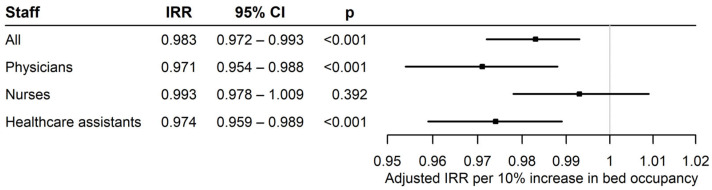
Multivariable analyses of the association between bed occupancy rate and hand hygiene performance for each group of staff members. The grey vertical line represents the null effect, i.e., no association between bed occupancy rate and hand hygiene performance. Each black square shows the estimated Incidence Rate Ratio (IRR) for a specific staff group, and the horizontal whiskers represent the 95% Confidence Intervals (CIs).

The total compliance for hand hygiene performance was 9966 (78.3%), with physicians accounting for 76.2% of their hand hygiene opportunities, nurses for 80.7%, and healthcare assistants for 76.9% (physicians vs. nurses *p* < 0.001, physicians vs. healthcare assistants *p* = 0.602, and nurses vs. healthcare assistants *p* = 0.092). The univariate analysis revealed that a higher bed occupancy rate was associated with lower hand hygiene performance (a 10% increase in bed occupancy was associated with a 1.3% decline in staff compliance, *p* = 0.001). However, when analyzed separately, there was a significant decline only among physicians (physicians: 2.1%, *p* < 0.001; nurses: 0.05%, *p* = 0.053; healthcare assistants: 1.7%, *p* = 0.07). The multivariable analysis showed that a higher bed occupancy rate was also significantly associated with lower hand hygiene performance (adjusted IRR 0.983, 95%CI 0.972–0.993, *p* < 0.001). There was a significant decline among physicians (adjusted IRR 0.971, 95%CI 0.954–0.988, *p* < 0.001) and healthcare assistants (adjusted IRR 0.974, 95%CI 0.959–0.989, *p* < 0.001), but not among nurses (adjusted IRR 0.993, 95%CI 0.978–1.009, *p* = 0.392). Although no statistically significant differences were observed between staff categories, there was a trend suggesting that physicians and healthcare assistants exhibited a slightly greater decline in hand hygiene compliance compared with nurses (physicians vs. nurses *p* = 0.061, nurses vs. healthcare assistants *p* = 0.084, physicians vs. healthcare assistants *p* = 0.795). The univariate and multivariable analyses are summarized in Table 2 and Figure 1. Weekday/weekend, sex, age, proportion of ventilated patients, the presence of comorbidities, and patient functional status were not significantly associated with hand hygiene performance.

Further analysis showed that after controlling for confounders, a bed occupancy rate of ≥110% was associated with significantly lower hand hygiene compliance (IRR = 0.949, 95%CI 0.921–0.977, *p* < 0.001) compared to a bed occupancy rate of <100%. Bed occupancy rate of 100–109% also showed lower hand hygiene compliance; however, it did not reach statistical significance (IRR = 0.975, 95%CI 0.937–1.016, *p* = 0.289).

## 4. Discussion

The increase in life expectancy worldwide has led to a higher rate of hospitalization, and the patients are more likely to be characterized by older age and serious medical conditions [1,2,3], leading to an increase in the bed occupancy rate in the internal medicine departments [2,4,5,6]. Previous studies have shown an association between bed occupancy rate and quality of care as measured by quality indexes [7]. Adherence to hand hygiene is one of the main measures in hospitals. Hand hygiene plays an important role in preventing the transmission of pathogenic microorganisms to patients and healthcare workers [9,10,11,12,13]. To the best of our knowledge, associations between bed occupancy rate and the performance of hand hygiene by the staff in internal medicine departments have not been previously investigated.

Several studies tried to identify risk factors for nonadherence of healthcare workers to required hand hygiene practices. Both Pittet et al. and Moore et al. demonstrated that a high workload is associated with increased risk for nonadherence [14,17], and Chang et al. found that compliance was significantly lower during high workload periods [28]. Those authors also showed that hand hygiene compliance remained stable until the workload approached 30 hand hygiene opportunities per hour, after which compliance decreased significantly. A study conducted by Le et al., which aimed to identify barriers to compliance with hand hygiene protocols among medical staff, observed that patient overcrowding was among the main barriers to compliance [15]. Similar results were reported by Carter et al.’s study conducted in the ED setting, in which hand hygiene compliance was significantly lower when it was heavily crowded [13]. Clements et al. found that overcrowding and understaffing in hospitals lead to a decrease in compliance with hand hygiene by healthcare workers, which leads to a failure in controlling MRSA infections [16].

In the present study, we aimed to evaluate the association between bed occupancy rate and staff compliance with hand hygiene protocol in internal medicine departments. Our ecological study included 12,736 hand hygiene opportunities. The total hand hygiene performance rate was 78.3%. This rate was significantly lower among physicians than among nurses. These results are consistent with previous studies [15,28,29]. We also showed that an increase of 10% in bed occupancy rate was associated with an approximately 2% decline in staff compliance. Our findings are in agreement with those of other reports [13,14,15,16,17,28]. Separate analysis of each of the 3 groups that comprised the staff showed a significant decline solely among healthcare assistants, as well as a similar trend among nurses that did not reach a level of significance. Nurses likely demonstrated higher hand hygiene compliance overall. This may be explained by their more comprehensive training in hand hygiene and their frequent direct patient contact, which increases both the number of hand hygiene opportunities and the perceived importance of adherence for patient safety [30,31]. Qualitative studies further suggest that infection prevention is viewed by nurses as a core professional responsibility, reinforced early in training and sustained through workplace social norms and peer expectation [30,32].

According to previous studies, the negative impact of high bed occupancy on hand hygiene compliance is primarily mediated by increased workload, time constraints, and staff fatigue. Overcrowding and understaffing lead to a greater number of hand hygiene opportunities per hour, which in turn reduces the likelihood that staff will perform hand hygiene as recommended [10,16,17]. Staffing levels in the departments were fixed across all sectors (physicians, nurses, healthcare assistants) and did not vary according to workload. Hence, the study assumed that staffing levels would be unfavorable during periods of high occupancy. An occupancy rate above 100% indicates overcrowding, whereby patients are placed in areas not intended for patient care (e.g., hallways), where hand hygiene materials are limited (such as sinks), and, consequently, hand hygiene compliance may be reduced. However, in our setting, point-of-care alcohol-based hand rub is available on each patient bed.

Poor hand hygiene performance is associated with hospital-acquired infections (HAI), and HAI are associated with extending the length of stay, poor clinical outcomes (including deaths), and higher hospital costs [33,34]. Hand hygiene compliance is particularly critical in internal medicine departments, where a high proportion of patients are elderly, frail, and immunocompromised, making them more susceptible to harm from HAIs [10]. There is a globally increasing trend of increased workload and rising rates of hospital-acquired infections [35,36,37]. The association we observed between bed occupancy rates and staff hand hygiene compliance may inform the development of infection prevention strategies across diverse healthcare systems and hospital administrators. Such strategies may include targeted hand hygiene education focusing on physicians and healthcare assistants (given that we observed no association between bed occupancy and hand hygiene among nurses), increasing staff-to-bed ratios, and implementing alternative care models, such as telemedicine, long-term care facilities, and home-based hospitalization programs.

Our study has several limitations. First, its ecological design precludes individual study of the individual. Second, data from 2017 to 2019 were used in order to avoid any influence of the COVID-19 pandemic waves on the study results, and future studies will be needed to confirm the results. Third, the study was conducted at a large tertiary medical center and therefore may not represent community hospitals. Fourth, the investigation was limited to internal medicine departments, and the findings may not be applicable to other departments. Fifth, the overt observations of compliance with hand hygiene performance may have stimulated greater compliance than if those observations had been covert, which is known as the Hawthorne effect [38]. However, the Hawthorne effect is known to be attenuated under conditions of increased workload [39]. Therefore, our findings likely provide a reasonable reflection of actual hand hygiene performance, particularly in high-occupancy situations. Sixth, additional confounders such as staffing ratios, shift times, or seasonal effects were not included in the analysis. Although staffing levels vary between shifts, these fluctuations do not substantially change throughout the day, and we assume no meaningful seasonal variation in hand hygiene compliance. Seventh, due to the historical nature of the study, information on inter-rater reliability of the observers was unavailable. Eighth, hand hygiene assessments were conducted mostly on weekdays, resulting in negligible representation of weekends. Therefore, further studies will be needed to assess the association between hand hygiene compliance and bed occupancy during weekends. Ninth, the current study did not assess HAI rates during the study period; future research could explore the relationship between bed occupancy and HAI rates. Tenth, patient isolation status was not stored routinely in the medical records, and therefore, we cannot rule out the impact on hand hygiene compliance. Lastly, although other measures for workload may be available, especially in prospective studies, bed occupancy is easy to estimate, and the data are readily available. Future studies will be needed to explore the association with other workload indices.

Our study has several strengths. First, this study was conducted in a large tertiary medical center comprising 9 internal medicine departments with an average of 300 beds, which enabled the collection of a substantial dataset of 12,736 hand hygiene opportunities over a three-year period. Such a large number of observations is uncommon in previous studies, which typically report only 800–2000 opportunities and are rarely conducted exclusively within internal medicine departments [13,14,15,16,17]. Second, data were collected from electronic medical records and the institutional database, both of which are used for patient management on a daily basis. Third, hand hygiene performance was evaluated by qualified and experienced observers and reported to the Ministry of Health as part of regularly conducted quality index surveys. Fourth, the level of compliance was evaluated according to the “Five Moments” model, which is well established in the literature and part of WHO guidelines.

## 5. Conclusions

The results of our study show that an increase in bed occupancy rate is associated with a decrease in compliance with hand hygiene regulations on the part of the staff of internal medicine departments. Steps to improve hand hygiene compliance—such as enhancing hand hygiene education, increasing staff-to-bed ratios, and utilizing out-of-hospital facilities—are essential to prevent negative clinical outcomes, including hospital-acquired infections.

## Figures and Tables

**Table 1:** Department characteristics on the days of observation.

Characteristics	
Number of days	1043
Bed occupancy rate (%), mean (SD)	99.0 (14.2)
Females (%), mean (SD)	46.8 (8.9)
Average age (years), mean (SD)	71.6 (3.1)
Rate of ventilated patients (%), median (IQR)	8.2 (5.4–10.8)
Norton scale, median (IQR)	17.2 (16.0–19.0)
Charlson Comorbidity Index, median (IQR)	4.0 (3.0–5.0)
Weekend, n (%)	15 (1.4%)

**Table 2:** Univariate and Multivariable Analyses of the Association between Bed Occupancy Rate and Hand Hygiene Performance.

Staff	Type	Variable	IRR (95%CI)	*p*
All	Univariate	Bed occupancy rate (10%)	0.987 (0.978–0.995)	0.001
	Multivariable	Bed occupancy rate (10%)	0.983 (0.972–0.993)	<0.001
		Female rate (10%)	0.992 (0.968–1.017)	0.527
		Average age (10 years)	0.967 (0.910–1.028)	0.284
		Weekend	1.023 (0.911–1.149)	0.702
		Ventilated patients rate (%)	0.996 (0.991–1.000)	0.064
		Norton scale	0.999 (0.991–1.007)	0.777
		Charlson Comorbidity Index	0.993 (0.984–1.003)	0.193
Physicians	Univariate	Bed occupancy rate (10%)	0.979 (0.967–0.99)	<0.001
	Multivariable	Bed occupancy rate (10%)	0.971 (0.954–0.988)	<0.001
		Female rate (10%)	0.979 (0.949–1.010)	0.185
		Average age (10 years)	0.968 (0.894–1.047)	0.413
		Weekend	1.022 (0.883–1.183)	0.771
		Ventilated patients rate (%)	0.998 (0.994–1.003)	0.403
		Norton scale	1.001 (0.988–1.015)	0.827
		Charlson Comorbidity Index	1.006 (0.988–1.024)	0.506
Nurses	Univariate	Bed occupancy rate (10%)	0.995 (0.979–1.012)	0.553
	Multivariable	Bed occupancy rate (10%)	0.993 (0.978–1.009)	0.392
		Female rate (10%)	1.015 (0.993–1.038)	0.187
		Average age (10 years)	0.936 (0.885–0.990)	0.021
		Weekend	1.018 (0.957–1.083)	0.571
		Ventilated patients rate (%)	0.998 (0.994–1.002)	0.355
		Norton scale	0.998 (0.992–1.005)	0.581
		Charlson Comorbidity Index	0.998 (0.985–1.012)	0.771
Healthcare assistants	Univariate	Bed occupancy rate (10%)	0.983 (0.964–1.002)	0.074
	Multivariable	Bed occupancy rate (10%)	0.974 (0.959–0.989)	<0.001
		Female rate (10%)	0.975 (0.929–1.023)	0.305
		Average age (10 years)	0.944 (0.852–1.045)	0.266
		Weekend	1.081 (1.025–1.139)	0.004
		Ventilated patients’ rate (%)	0.993 (0.985–1.001)	0.103
		Norton scale	1.001 (0.984–1.019)	0.881
		Charlson Comorbidity Index	1.008 (0.985–1.032)	0.482

## Data Availability

Due to privacy limitations imposed by our IRB, the data supporting the findings of this study are available from Prof. Gil Fire, upon reasonable request and subject to approval by the institutional review board.

## Abbreviations

The following abbreviations are used in this manuscript:

CCI	Charlson Comorbidity Index
GEE	Generalized Estimating Equation
IRR	Incidence Relative Ratio
ED	Emergency Departments
